# *Candida auris* Whole-Genome Sequence Benchmark Dataset for Phylogenomic Pipelines

**DOI:** 10.3390/jof7030214

**Published:** 2021-03-16

**Authors:** Rory M. Welsh, Elizabeth Misas, Kaitlin Forsberg, Meghan Lyman, Nancy A. Chow

**Affiliations:** Mycotic Diseases Branch, Centers for Disease Control and Prevention, Atlanta, GA 30333, USA; nye9@cdc.gov (E.M.); lnv6@cdc.gov (K.F.); yeo4@cdc.gov (M.L.); yln3@cdc.gov (N.A.C.)

**Keywords:** *Candida auris*, emerging fungal diseases, drug-resistant fungi, genomic, whole-genome sequencing WGS

## Abstract

*Candida auris* is a multidrug-resistant pathogen that represents a serious public health threat due to its rapid global emergence, increasing incidence of healthcare-associated outbreaks, and high rates of antifungal resistance. Whole-genome sequencing and genomic surveillance have the potential to bolster *C. auris* surveillance networks moving forward. Laboratories conducting genomic surveillance need to be able to compare analyses from various national and international surveillance partners to ensure that results are mutually trusted and understood. Therefore, we established an empirical outbreak benchmark dataset consisting of 23 *C. auris* genomes to help validate comparisons of genomic analyses and facilitate communication among surveillance networks. Our outbreak benchmark dataset represents a polyclonal phylogeny with three subclades. The genomes in this dataset are from well-vetted studies that are supported by multiple lines of evidence, which demonstrate that the whole-genome sequencing data, phylogenetic tree, and epidemiological data are all in agreement. This *C. auris* benchmark set allows for standardized comparisons of phylogenomic pipelines, ultimately promoting effective *C. auris* collaborations.

## 1. Introduction

The emerging multidrug-resistant pathogenic yeast *Candida auris* has been reported in over 40 countries and represents a threat to global health [1,2]. Patient-to-patient spread has been documented in multiple countries [3]. Infection prevention guided by rapid detection in healthcare settings is essential because *C. auris* is easily spread in healthcare settings as it colonizes patients’ skin and can survive and persist in the clinical environment for weeks [4]. *C. auris* is capable of causing severe bloodstream infections and has become the leading cause of invasive candidiasis in some hospitals [5]. In addition, *C. auris* is difficult to treat as it is commonly resistant to multiple antifungal drug classes.

Whole-genome sequencing (WGS) based methods have increasingly been used to detect and characterize outbreaks for this emerging pathogen [2,6,7]. Population analysis of *C. auris* genomic data has revealed that most of the detected cases are stratified into four major clades (Clades I, II, III, and IV) except for a single representative case describing a potential fifth clade (Clade V) [8,9]. Currently, WGS is used as part of the public health response to halt the spread of *C. auris* by helping to characterize introductions, phylogeographic mixing, and transmission dynamics [2]. The potential for genomic surveillance to serve as a powerful tool to support epidemiological investigation in public health is clear, but genomic analysis methods need to be validated and documented as a prerequisite for public health authorities to trust genomic data in routine investigations [10].

As access to genomic sequence data has grown to help meet the challenge of investigating transmission chains and large-scale pathogen populations, the capacity to analyze the ever-increasing amount of sequence data has struggled to keep up. One common bottleneck of scaling analysis capacity often occurs when onboarding and validating new and constantly changing phylogenomic pipelines and methods to identify variants and distinguish related genomes [11]. Previous studies have utilized single nucleotide polymorphisms (SNPs), short tandem repeat, and multilocus sequence typing (MLST) strategies for molecular typing of *C. auris* outbreaks [12,13]. Outbreak datasets with publicly available raw reads have been published for multiple outbreak events in North America and globally [6,14]. However, a standardized dataset to allow for comparisons of these molecular typing and phylogenomic pipelines has not been established for *C. auris*. Therefore, we set out to establish a genomic benchmark dataset to serve as a resource to facilitate global efforts to collaborate and rapidly validate sequence analysis tools.

Here, we present an empirically and epidemiologically validated outbreak dataset of Clade I isolates from North America in a standardized format that enables easy access and automated analysis. To ensure computational feasibility on a wide range of computing infrastructures, we included a small subset of these well-vetted genome sequences in the *C. auris* benchmark dataset. This resource provides an important first step towards a collaborative infrastructure for academic and public health authorities to document and validate variant calling and resulting phylogenetic tree topologies to aid communication, outbreak genomic surveillance, and containment efforts.

## 2. Materials and Methods

The *C. auris* isolates used in this study were from a subset of outbreak cases from the United States where the sequence data and epidemiological data, such as the facility where the patient isolates were collected, are all in agreement as previously described by [2,6]. The isolates included in the study belong to the two Clade I lineages involved in outbreaks (subclade 1b and 1c), the complete reference genome is from subclade 1c, and the subclade designations were previously described (Muñoz et al. 2021). The isolates were obtained from clinical and colonization cases, as defined by The Council of State and Territorial Epidemiologists (CSTE). For quality assurance, sequences with greater than 20× sequencing coverage were selected for the dataset, which is the quality threshold average coverage guideline for the National Center for Biotechnology Information (NCBI) Pathogen Detection. Strain identifiers for each isolate, accession numbers to the genomes, subclade, PubMed study identifiers, and outbreak and facility anonymized codes are listed in Table 1. The sequencing metrics (sequence quality, total reads, assembly length, and contigs) for each genome were obtained from NCBI [15]. Additionally, we used FastQC to estimate the sequence read quality and percent GC content [16]. We identified SNPs using MycoSNP GeneFlow workflows (https://git.biotech.cdc.gov/geneflow-workflows/mycosnp/), which performs read mapping using Burrows-Wheeler Aligner (BWA) v0.7.17, SNP calling using Genome Analysis Toolkit (GATK) v4.1.4.1, and generates a multi-FASTA file of the informative variants. To ensure that multiple phylogenetic analyses produce similar results, maximum parsimony and maximum likelihood phylogenetic analyses with 1000 bootstraps using MEGA version X were generated [17] as previously described [6] and visualized on Microreact [18]. All of sequence data and tree materials are publicly available for download at GitHub: https://github.com/globalmicrobialidentifier-WG3/datasets (accessed on 18 February 2021).

## 3. Results

**Figure 1:**
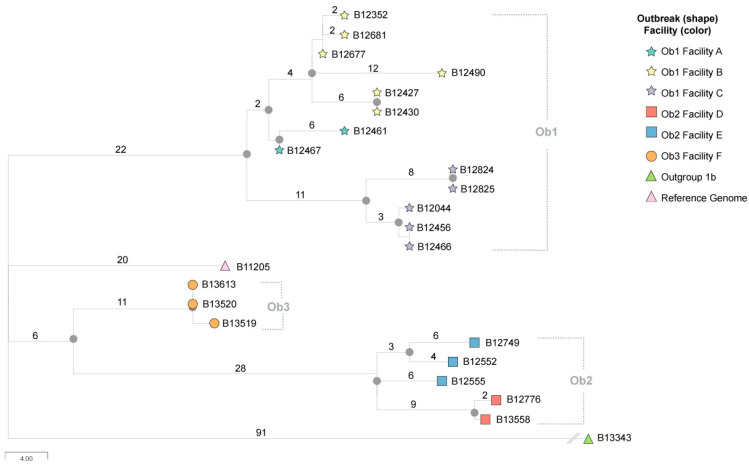
Phylogeny of *Candida auris* benchmark dataset including cases from Clade I. Numbers above the branch indicate the number of changes that have occurred in that branch length, and the internal solid circle nodes indicate separations with a bootstrap value of at least 90%. Each leaf node represents a unique isolate; the shape of the node refers to the outbreak number code and the node color refers to the facility.

Twenty-four isolates from 21 clinical or colonization *C. auris* cases were included in this dataset. These cases were reported during 2016–2017 United States regional spread where ongoing transmission was previously described for healthcare facilities in New York, New Jersey, and Massachusetts [6]. Specifically, three Clade I outbreaks (Outbreak 1–3) that each form their own separate monophyletic branch and comprise eight healthcare facilities (Facility A–G) are represented in this dataset (Figure 1). Clade I includes isolates representing three major lineages; two lineages are routinely involved in outbreaks and are diverged from a small subclade that includes the commonly used B8441 reference genome (Muñoz et al. 2021). These were previously designated subclade 1a, 1b, and 1c and representatives of each subclade are contained in the dataset (Table 1). From previous descriptions, subclade 1a includes the B8441 reference, subclade 1b contains isolates from cases in India, Pakistan, Kenya, France, Germany, China, United Kingdom, Saudi Arabia, and the United States (California and Connecticut); and subclade 1c contains isolates from cases in India, Pakistan, Saudi Arabia, and the United States (New York, New Jersey, and Massachusetts).

Phylogenetic analysis showed that all 23 genomes clustered with Clade I, with 22 (92%) belonging to subclade 1c, which represents three distinct New York, New Jersey, and Massachusetts outbreak branches (Figure 1). Of the subclade 1c isolates, 13 (59%) clustered with the New York outbreak 1 branch node, 5 (27%) form the New Jersey outbreak 2 branch node, and 3 (14%) with the Massachusetts outbreak 3 branch node (Figure 1). Outbreak 1 describes 13 genomes collected from 11 New York cases originating from three healthcare facilities (Facility A, B, and C) (Table 1). Outbreak 2 describes six genomes collected from five New Jersey cases in two separate healthcare facilities (Facility D and E). Outbreak 3 describes three genomes from three Massachusetts cases from a single facility (Facility F). The mean SNP difference among isolates obtained from outbreak codes 1, 2, and 3 were 16 (range 0–34), 15 (range 3–20), and 1 (range 0–2), respectively.

The single outgroup subclade 1b representative genome from a Connecticut case is clearly separated by >100 SNPs from the subclade 1c lineage genomes. Multiple independent phylogenetic analyses support the observed phylogenetic relationships, and Supplementary Figure S1 shows a maximum likelihood phylogeny with 1000 bootstrap replications produced similar results. The quality metrics for each genome are reported in Table 2. The scripts for downloading and accessing the associated benchmark files are available on GitHub: https://github.com/globalmicrobialidentifier-WG3/datasets (accessed on 18 February 2021).

## 4. Discussion

The *C. auris* empirical outbreak benchmark dataset was established to help standardize comparisons of genomic analysis tools designed for the specific purpose of validating molecular typing methods to aid outbreak surveillance. Compared to simulated datasets, manually established empirical benchmark datasets are often slow and tedious to generate, but they are a powerful resource for validating phylogenomic analysis tools (Timme et al. 2019). We included only a subset of the total cases from the three state outbreaks in our analysis; therefore, this dataset is not representative of the current *C. auris* molecular epidemiology observed in the United States. This resource represents well-vetted sequence data that can be used to compare test results against known results to aid validation efforts.

Limitations to consider for comparing SNP analyses are that the input sequence quality and coverage can generate different SNP numbers, which highlights the importance of adhering to sequence quality levels. Different sequence assembly tools, read mapping tools, and SNP pipelines can generate different SNP counts, but the topology should remain consistent. In addition, the number of SNPs described within an outbreak or healthcare facility is context dependent and may not be directly comparable with other studies due to multiple factors. For example, *C. auris* can colonize patients for extended periods of time (months to years) and coupled with the length of time for the on-going outbreak [19], the number of clades, number of importation events, and mixing can all expand the breath of genetic variation in an outbreak population.

*C. auris* is an emerging pathogen and more of these types of benchmark datasets will be needed. Future datasets should expand to include new clades, isolates, and scenarios to better serve the community. Benchmark datasets assembled through empirical approaches from well-vetted studies that are supported by multiple lines of evidence have been traditionally used for validation efforts [11,20]. Simulated datasets can allow for manipulation of multiple parameters and often compliment and help overcome some of the slow manual processes required for generating empirical dataset [21]. These rigorous phylogenomic validation practices and resources will help ensure confidence in utilizing various genomic analysis tools to inform public health decision and action. 

## Figures and Tables

**Table 1:** List of sequences included in the *Candida auris* whole-genome sequence benchmark dataset.

BioSample	Isolate ID	SRA Run	Outbreak ID	Facility ID	Subclade	Date Collected	Study *
SAMN10139509	B12352	SRR7909282	1	B	Ic	2016	30293877
SAMN10139317	B12681	SRR7909214	1	B	Ic	2017	30293877
SAMN10139320	B12677	SRR7909135	1	B	Ic	2017	30293877
SAMN10139376	B12490	SRR7909210	1	B	Ic	2016	30293877
SAMN10139493	B12427	SRR7909184	1	B	Ic	2016	30293877
SAMN10139490	B12430	SRR7909233	1	B	Ic	2016	30293877
SAMN10139465	B12461	SRR7909234	1	A	Ic	2016	30293877
SAMN10139461	B12467	SRR7909297	1	A	Ic	2016	30293877
SAMN10139268	B12824	SRR7909313	1	C	Ic	2017	30293877
SAMN10139267	B12825	SRR7909385	1	C	Ic	2017	30293877
SAMN10139529	B12044	SRR7909147	1	C	Ic	2016	30293877
SAMN10139470	B12456	SRR7909156	1	C	Ic	2016	30293877
SAMN10139462	B12466	SRR7909413	1	C	Ic	2016	30293877
SAMN10139189	B13613	SRR7909284	3	F	Ic	2017	30293877
SAMN10139194	B13520	SRR7909394	3	F	Ic	2017	30293877
SAMN10139195	B13519	SRR7909408	3	F	Ic	2017	30293877
SAMN10139295	B12749	SRR7909166	2	E	Ic	2017	30293877
SAMN10139364	B12552	SRR7909192	2	E	Ic	2017	30293877
SAMN10139361	B12555	SRR7909183	2	E	Ic	2017	30293877
SAMN10139289	B12776	SRR7909308	2	D	Ic	2017	30293877
SAMN10139190	B13558	SRR7909246	2	D	Ic	2017	30293877
SAMN10142006	B13343	SRR7909249	NA	G	Ib	2017	30293877

* PubMed Study Identifier.

**Table 2:** *Candida auris* whole-genome sequence benchmark dataset sequence metrics.

BioSample	Isolate ID	SRA Run	Total Reads (Millions)	Avg. GC Content (%)	Assembly Length	Contigs	N50
SAMN10139509	B12352	SRR7909282	3.4	45%	12,219,463	997	20,257
SAMN10139317	B12681	SRR7909214	6.2	47%	11,914,250	1030	19,891
SAMN10139320	B12677	SRR7909135	6.2	46%	12,125,777	1006	20,610
SAMN10139376	B12490	SRR7909210	3.6	43%	12,215,651	989	21,267
SAMN10139493	B12427	SRR7909184	2.8	42%	12,209,708	1030	20,335
SAMN10139490	B12430	SRR7909233	3.2	43%	12,212,626	990	20,510
SAMN10139465	B12461	SRR7909234	5.2	43%	12,223,488	1033	20,401
SAMN10139461	B12467	SRR7909297	5.4	44%	12,227,902	1049	20,090
SAMN10139268	B12824	SRR7909313	5	43%	12,217,316	1073	19,453
SAMN10139267	B12825	SRR7909385	6.4	44%	12,235,136	1011	21,091
SAMN10139529	B12044	SRR7909147	3.2	43%	12,219,487	979	21,004
SAMN10139470	B12456	SRR7909156	4.4	43%	12,218,763	1049	19,233
SAMN10139462	B12466	SRR7909413	5	43%	12,223,757	1038	19,885
SAMN10139189	B13613	SRR7909284	4.8	43%	12,225,913	976	21,224
SAMN10139194	B13520	SRR7909394	4.2	43%	12,215,973	1016	20,348
SAMN10139195	B13519	SRR7909408	2.8	44%	12,161,014	1039	20,040
SAMN10139295	B12749	SRR7909166	7.6	44%	12,234,239	993	21,085
SAMN10139364	B12552	SRR7909192	4.6	43%	12,217,679	983	21,519
SAMN10139361	B12555	SRR7909183	3.6	43%	12,215,609	982	21,427
SAMN10139289	B12776	SRR7909308	6.8	42%	12,228,293	989	21,510
SAMN10139190	B13558	SRR7909246	5.2	44%	12,222,520	982	21,174
SAMN10142006	B13343	SRR7909249	4.2	41%	12,213,890	997	21,547

## Data Availability

All of materials for the benchmark dataset are publicly available for download at GitHub: https://github.com/globalmicrobialidentifier-WG3/datasets (accessed on 10 February 2021).

## Supplementary Materials

The following are available online at https://www.mdpi.com/2309-608X/7/3/214/s1, Figure S1: Phylogeny of Candida auris benchmark dataset including cases from Clade I.
