# Supplementary material for: Candida auris Whole-Genome Sequence Benchmark Dataset for Phylogenomic Pipelines
Source: J Fungi (Basel). 2021 Mar 16;7(3):214. doi: 10.3390/jof7030214 (PMC8002225; doi:10.3390/jof7030214)
Supplement: Supplementary file 1 [file jof-07-00214-s001.pdf]

Supplementary File

Supplementary Figure 1. Phylogeny of *Candida auris* benchmark dataset including cases from Clade I. The internal solid circle nodes indicate separations with a bootstrap value of at least 90%, and the number above the branch indicate branch length. Each leaf node represents a unique isolate; the shape of the node refers to the outbreak number code and the node color refers to the facility.

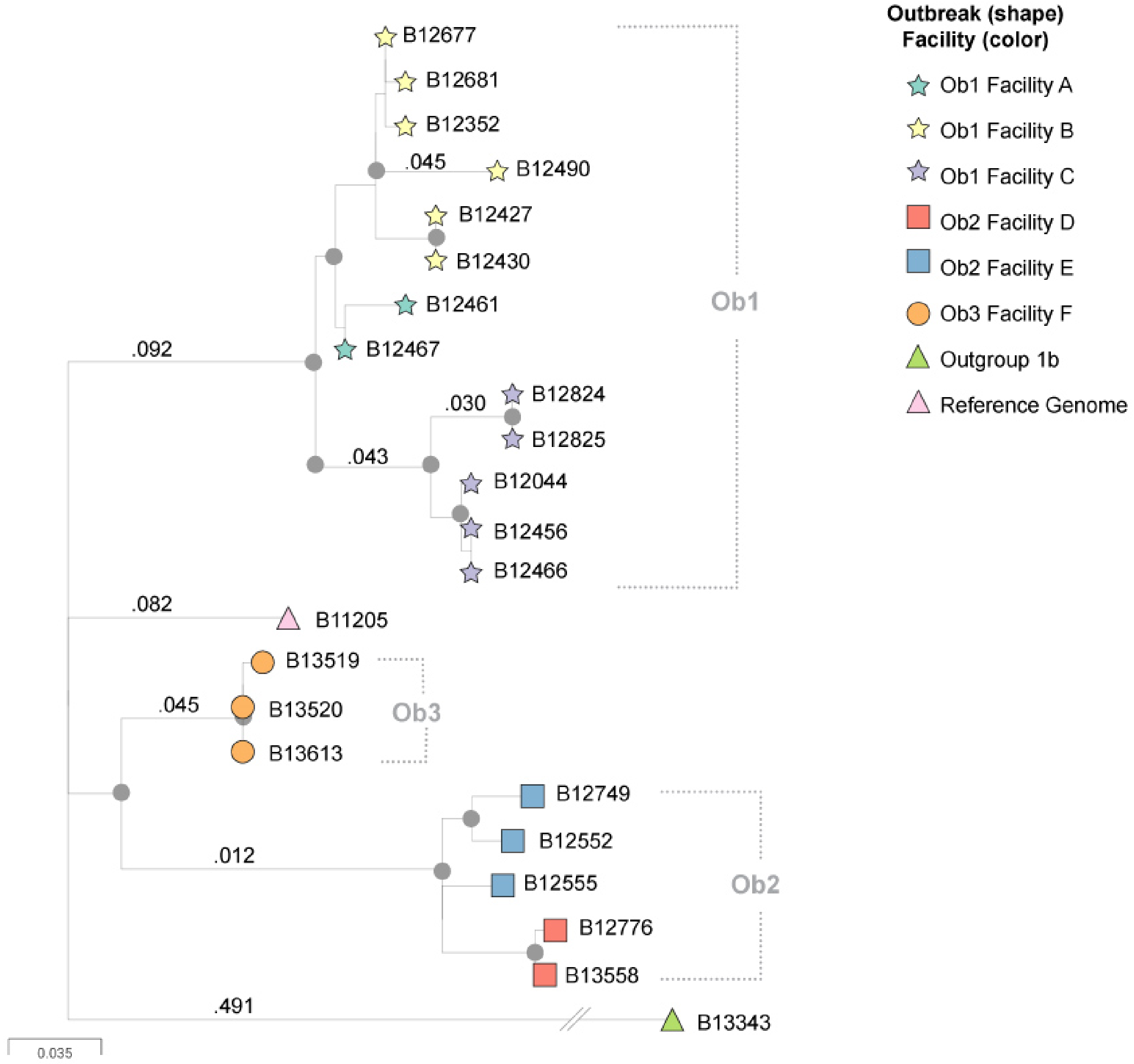

Figure 1. Phylogeny of *Candida auris* benchmark dataset including cases from Clade I. The internal solid circle nodes indicate separations with a bootstrap value of at least 90%, and the number above the branch indicate branch length. Each leaf node represents a unique isolate; the shape of the node refers to the outbreak number code and the node color refers to the facility.

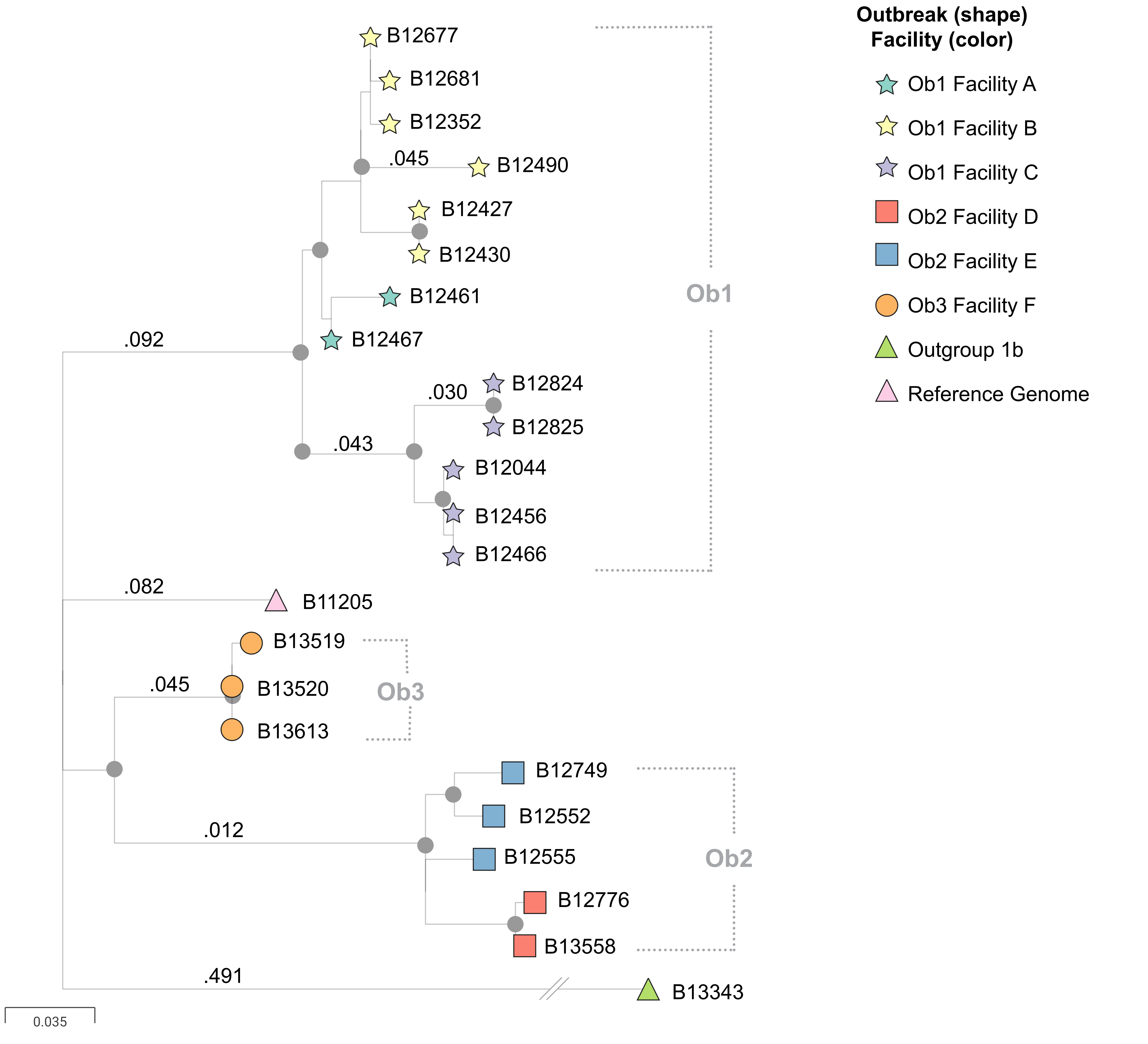

Outbreak (shape)
Facility (color)
Ob1 Facility A
Ob1 Facility B
Ob1 Facility C
Ob2 Facility D
Ob2 Facility E
Ob3 Facility F
Outgroup 1b
Reference Genome
B12677
B12681
B12352
.045
B12490
B12427
B12430
B12461
B12467
.092
Ob1
.030
B12824
B12825
.043
B12044
B12456
B12466
.082
B11205
B13519
.045
B13520
B13613
Ob3
B12749
B12552
.012
B12555
Ob2
B12776
B13558
.491
B13343
0.035
